# Epidemiology of Peste des Petits Ruminants in Nigeria: A Review

**DOI:** 10.3389/fvets.2022.898485

**Published:** 2022-07-06

**Authors:** Daniel Esonu, Bryony Armson, Mohammed Babashani, Ruth Alafiatayo, Abel B. Ekiri, Alasdair J. C. Cook

**Affiliations:** ^1^Department of Veterinary Public Health and Preventive Medicine, Faculty of Veterinary Medicine, Ahmadu Bello University, Zaria, Nigeria; ^2^vHive, Department of Veterinary Epidemiology and Public Health, School of Veterinary Medicine, University of Surrey, Guildford, United Kingdom

**Keywords:** PPRV, seroprevalence, vaccination, peste des petits ruminants, small ruminants, sheep, goats, camels

## Abstract

Peste des petits ruminants (PPR) is a major constraint to the productivity of small ruminants in Nigeria. Understanding of the current epidemiological status of PPR is crucial to its effective control. A review of the epidemiology of PPR in Nigeria was performed and research gaps were identified. Thirty-seven eligible articles were reviewed: these presented information from 30 of the 36 states of Nigeria. Most studies focused on goats and/or sheep (*n* = 33) but camels (*n* = 4), cattle (*n* = 1) and wild ruminants (*n* = 2) were also considered. Fourteen (37.8%) of the articles reported seroprevalence in small ruminants, which varied from 0.0% to 77.5% where more than 10 animals were sampled. Molecular characterization and phylogenetic analysis were performed in 6 studies, with lineages II and IV, detected in sheep and goats. In one study in small ruminants, sequences clustering into lineage I showed a similarity to the vaccine strain, Nigeria 75/1, based on phylogenetic analysis of *F* gene sequences. However, if the preferred method of sequencing the *N* gene had been performed, this isolate would have been grouped into lineage II. According to *N* gene phylogenetic analysis in the other studies, sequences were identified that clustered with clade II-NigA, II-NigB (closely related to the Nigeria 75/1 vaccine strain), and others which were well separated, suggesting a high diversity of PPRV in Nigeria. Five articles reported the detection of lineage IV in 22/36 states, with IV-NigA and IV-NigB detected, highlighting its widespread distribution in Nigeria. Risk factors for PPRV seropositivity were reported in 10/37 (27.0%) articles, with a higher seroprevalence observed in female animals, although differing results were observed when considering species and age separately. There were inconsistencies in study design and data reporting between studies which precluded conduct of a meta-analysis. Nevertheless, several research gaps were identified including the need to investigate the low uptake of PPRV vaccine, and the economic benefits of PPR control measures to small ruminant farmers. Such data will inform PPR control strategies in Nigeria and subsequently contribute to the global 2030 PPR eradication strategy.

## Introduction

Peste des petits ruminants (PPR) is a viral disease, caused by the small ruminant morbillivirus (SRMV, commonly termed as PPRV), a member of the *Morbillivirus* genus, in the family *Paramyxoviridae* (1). Based on genetic relationships between PPR viruses from different geographical regions, Shaila et al. (2) reported that PPRVs have undergone independent evolution which has resulted in four genetic subtypes (PPRV lineages I–IV). PPR is a disease of mainly goats and sheep with high morbidity and mortality that can reach 100 and 90%, respectively (3) and consequently is a major constraint to small ruminant production (4, 5). Evidence of PPRV infection has also been identified in camels (6, 7), cattle (8), buffaloes (9) and several wildlife species, however their role in PPR viral circulation has not been properly elucidated (10).

Clinical signs of PPR include fever, depression, serous ocular and nasal discharges, erosive lesions on mucous membranes, stomatitis, and gastroenteritis (11–13). In most fatal infections, death is caused by primary bronchopneumonia or severe dehydration caused by acute diarrhea (14, 15). Additionally, infection of pregnant animals has been linked to abortion (16). PPR results in financial challenges for smallholder farmers and stakeholders: direct financial losses due to PPR include mortality, lower reproductive capacity, and reduced milk production; whilst indirect financial losses include a reduction in the value of surviving animals, restrictions on movements and sales, and cost of control measures such as vaccination (17–19).

The Food and Agriculture Organization (FAO) and the World Organization for Animal Health (OIE) named PPR as one of the five most destructive transboundary diseases in Africa, Asia, and the Middle East for small ruminant production and poverty alleviation efforts (3). The economic losses caused by PPR range from 1.2 to 2.1 billion US dollars globally each year (3, 18). In 2015, a study estimated the expected annual loss due to PPR in India to range from 2 million to 18 million US dollars and models predicted this could go up to 1.5 billion US dollars (20). The annual losses due to PPR in Kenya were estimated to be over $15 million (21). In Nigeria, a report by the International Livestock Research Institute (22) estimated an annual loss caused by PPR to be 4.3 billion naira (10.4 million US dollars).

Peste des petits ruminants has been reported in most parts of Africa, the Arabian Peninsula, the Middle East, and in central and south-East Asia (2, 23, 24), affecting and threatening an increasing number of small ruminants and livestock (25). PPRV is currently believed to be endemic across most countries of West Africa (26), with PPRV strains from lineages I, II and IV having been reported, although many outbreaks are not characterized at the molecular level.

Peste des petits ruminants continues to pose a significant challenge to small ruminant farmers in Nigeria, reducing the potential for the small ruminant population to significantly contribute to the supply of animal protein needs of Nigerians (27, 28). In Nigeria, there is a dearth of information regarding the current epidemiological status of PPR, which is crucial to its effective control is crucial to its effective control. This systematic review aimed to summarize peer-reviewed literature published since 2000 on the epidemiology of PPR in Nigeria, including seroprevalence and molecular epidemiology and to identify research gaps. The findings from this review may inform the design and implementation of PPR control strategies which may subsequently contribute to the global 2030 PPR eradication efforts (3, 18).

## Methods

### Literature Search

Five online database platforms were used for the search: PubMed, Web of Science, African Journals Online and Science Direct. Google Scholar was also utilized after the initial search to identify any articles not covered by these databases. The search was performed in February 2021 and undertaken using the following search terms: “(peste des petits ruminants OR PPR) AND Nigeria AND (small ruminants OR sheep OR ovine OR goat OR caprine OR wildlife OR camel).”

### Eligibility Criteria

All studies reporting the prevalence, risk factors, diagnosis, prevention, and control of PPR in Nigeria and published in the English language between 2000 and 2021 were included in the review. Review articles, conference proceedings, and book chapters were excluded to ensure only original data was obtained that contained adequate detail. The Preferred Reporting Items for Systematic Reviews and Meta-Analyses (PRISMA) guidelines for identifying potential articles of relevance, assessing the relevance of the articles and data extraction were utilized for this review (29).

**Figure 1:**
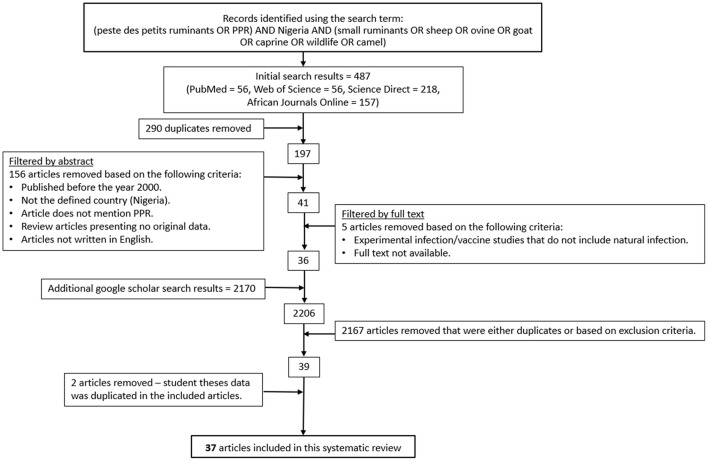
The process of literature search and eligible article selection.

The titles and abstracts of the retrieved articles were screened for eligibility based on the criteria detailed in Figure 1. For articles whose relevance could not be determined by reading the abstract alone, full texts were retrieved. Subsequently, a full text analysis for each remaining article was performed by two independent reviewers to further assess eligibility based on inclusion of at least one of the following: (i) a description of the study design, sampling strategy and approach, (ii) for prevalence studies, at least one appropriate diagnostic test was applied, (iii) for epidemiological studies the study population, sample size, number of cases, and study region were described, and (iv) for studies investigating risk factors for PPR, estimates of the strength of association were provided. Articles that did not meet at least one of the above criteria were excluded. Specific study designs were not excluded, and if an article met one criterion, but not another, data were used for the reported aspect (e.g., sero-prevalence data in an article was utilized; however, if the article also suggested risk factors that were not based on statistical evidence, then these were not used).

### Data Extraction

Data was extracted from eligible articles using a pre-designed Microsoft Excel data extraction form by DE, MB, and BA (Supplementary File 1). The type of data extracted included: title of the article, author(s), year, location of study, diagnostic test used, prevalence or sero-prevalence, and lineage detected.

### Data Analysis

Descriptive data analysis and figure preparation was performed using R (version 2021.09.1).

## Results

The initial database searches revealed 2,657 research articles before removing duplicates and those that did not meet the eligibility criteria, or where the full article could not be accessed. Two theses were excluded because the data were already presented in published journal articles that had been included in this study. Thirty-seven eligible articles were identified for data extraction and qualitative analysis (Figure 1). The studies were heterogeneous in terms of study design, animal subpopulations under investigation and sample size.

At least one study was performed in 30 of the 36 states of Nigeria. Two articles only reported the region and not the state(s) in which the research was conducted [South-East Nigeria (30) and the semi-arid region of North-eastern Nigeria (8)] (Table 1, Supplementary Table 1). Most studies were performed in Oyo State (*n* = 10) and Plateau State (*n* = 8). Many of the articles performed studies in multiple states. Delta, Edo, Ekiti, Jigawa, Nassarawa and Zamfara States and the Federal Capital Territory were not mentioned in any of the articles.

**Table 1:** The number of eligible articles where studies were performed per state.

**Geopolitical zone**	**State**	**Number of articles**	**Geopolitical zone**	**State**	**Number of articles**
NW	Jigawa	0	SE	Abia	2
NW	Kaduna	2	SE	Anambra	5
NW	Kano	6	SE	Ebonyi	2
NW	Katsina	2	SE	Enugu	4
NW	Kebbi	1	SE	Imo	5
NW	Sokoto	6	SE	Total	18
NW	Zamfara	0	SS	Akwa Ibom	5
NW	Total	17	SS	Bayelsa	2
NC	Benue	4	SS	Cross River	5
NC	Federal Capital Territory	0	SS	Delta	0
NC	Kogi	1	SS	Edo	0
NC	Kwara	4	SS	Rivers	2
NC	Nasarawa	0	SS	Total	14
NC	Niger	1	SW	Ekiti	0
NC	Plateau	8	SW	Lagos	1
NC	Total	18	SW	Ogun	3
NE	Adamawa	6	SW	Ondo	5
NE	Bauchi	4	SW	Osun	3
NE	Borno	6	SW	Oyo	10
NE	Gombe	1	SW	Total	22
NE	Taraba	4			
NE	Yobe	3			
NE	Total	24			

*NW, North West; NC, North Central; NE, North East; SE, South East; SS, South South; SW, South West*.

Most studies focused on small ruminants (either goats and/or sheep, *n* = 33), while some focused on camels (*n* = 4) (6–8, 31), cattle (*n* = 1) (8) and wild ruminants, including gazelle (*Dorcas gazelles*) (*n* = 1) (32) and African gray duiker (*Sylvicapra grimmia*) (*n* = 1) (33), either alone or in combination with small ruminants.

### Clinical Signs

Fourteen of the 37 articles (37.8%) (15, 34–46) described the clinical signs of PPR observed in outbreaks in Nigeria. These studies were all focused on sheep and/or goats and reported nasal discharges, swollen/crusty lips, conjunctivitis, coughing, dyspnea, diarrhea, pyrexia, depression, and anorexia. Also reported by at least one study were: erosions on the gums, necrosis of the dorsal surface of the tongue, whitish membranous covering all over the buccal cavity, corneal opacity, congestion and hepatization of the lungs, pneumonia, oral necrotizing and ulcerative stomatitis, congestion/distension in the large intestine and death in severe cases. Abortion was linked to PPR in three articles (35, 40, 47) and Wachida et al. (35) also reported infertility, stillbirth and mortality at weaning. Clinical signs were not reported in the papers that considered camels (6–8, 31).

Secondary or concurrent bacterial infections were described in two articles and included mannheimiosis (pneumonic pasturellosis) in PPRV-infected sheep and goats (41), and *Staphylococcus aureus* and streptococci in goats (40). Two articles described the co-infection of PPRV and goat pox virus in the same flock of sheep and goats in Plateau state. Vesicles around the mouth, generalized cutaneous pox lesions and mastitis were also observed in those studies (43, 44).

### Diagnosis

Based on the articles reviewed, several methods were used to identify previous or current PPR infection in Nigeria. A total of 18/37 (48.6%) articles determined sero-prevalence using serological tests including the competitive-enzyme-linked immunosorbent assay (cELISA) directed against the hemagglutinin (*H*) antigen (*n* = 8) (6–8, 32, 33, 36, 45, 48) or nucleoprotein (*N*) (*n* = 6) (31, 34, 44, 47, 49, 50) of the PPR virus, the virus neutralization test (VNT; *n* = 2) (8, 15) and/or the hemagglutination-inhibition (HI) test (*n* = 1) (46) (Supplementary Table 1). One study utilized both the VNT and cELISA for determination of seroprevalence (8), and two studies used an ELISA but did not provide specific details regarding the type of test or target protein (35, 51).

Some studies (*n* = 7) used conventional viral detection tests such as virus isolation (*n* = 2) (15, 46), agar gel immunodiffusion (AGID) (*n* = 3) (15, 52, 53), haemagglutination (HA) test (*n* = 1) (54), immunocapture ELISA (ICE) (*n* = 1) (31), immunohistochemical detection (*n* = 1) (55). Other studies used reports of clinical history (*n* = 14) (32, 38–41, 44–46, 53, 54, 56–59) and/or post-mortem observations (*n* = 10) (15, 40, 41, 44–46, 53, 55, 56, 59), either alone or in addition to diagnostic tests (Supplementary Table 1). One study used a combination of histopathology and transmission electron microscopy following necropsy (43). Three studies (35, 60, 61) also performed a structured questionnaire targeting goat farmers to determine the perceived occurrence and history of PPR in their herds.

Additionally, eight studies utilized molecular diagnostics for PPRV (30, 37–39, 42, 44, 58, 62). These studies reported PPRV results using the reverse transcription polymerase chain reaction (RT-PCR), targeting the PPRV nucleoprotein (*N*) gene (*n* = 7) (30, 37–39, 42, 44, 58) and the PPRV fusion gene (*F*) gene (*n* = 1) (62), and sequencing (*n* = 7) (30, 37–39, 42, 44, 62) (Supplementary Table 1). Chukwudi et al. (30) also reported results from colorimetric loop-mediated isothermal amplification (cLAMP) in addition to RT-PCR.

### Seroprevalence

**Figure 2:**
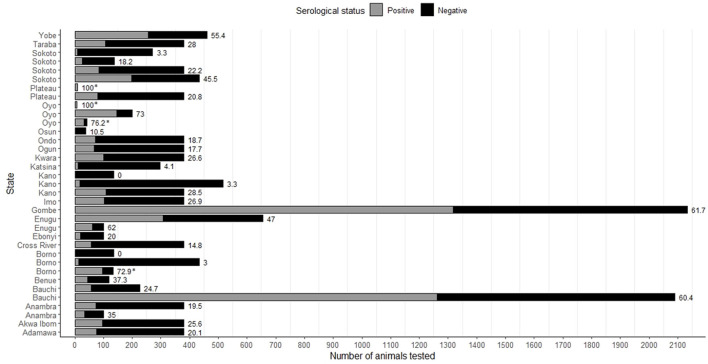
Seroprevalence of PPRV in all species considered, as reported by state of Nigeria in the eligible articles. In some states, more than one study was performed. Bar labels represent the seroprevalence as a percentage for that study/state. Asterisks indicate where the seroprevalence estimate is linked to a PPRV outbreak.

As mentioned previously, 18 articles determined PPRV sero-prevalence using serological tests (Supplementary Table 1). Data extracted from the eligible articles revealed that at least one seroprevalence study was conducted in each of the six different geopolitical zones of Nigeria within the past 21 years, and in 22 of the 36 states of Nigeria (Figure 2). Considering all species, the North East geopolitical zone had the most reports of PPR seroprevalence (9/18, 50.0%), while the South South zone (2/18, 11.1%) had the least. In 3/18 (16.7%) articles (31, 34, 44) the seroprevalence studies were conducted across at least two different geopolitical zones. A summary of seroprevalence estimates by geopolitical zone for all species is shown in Table 2. The number of animals sampled by location, sex and species are listed in the Supplementary Table 1, for those articles that provided this information.

**Table 2:** Summary of seroprevalence estimates for all species by geopolitical zone.

**Geopolitical zone**	**Number of studies**	**Seroprevalence (min/median/max reported values)**		
		**Min (%)**	**Median (%)**	**Max (%)**
North West	8	0.0	11.2	45.5
North East	9	0.0	29.5	72.9
North Central	4	21.4	32.7	100.0
South West	6	10.5	46.0	100.0
South East	6	20.3	31.6	62.2
South South	2	14.5	20.15	25.9

A total of 4/18 of the seroprevalence studies were linked to outbreak investigations that took place as part of the same study (15, 36, 44, 45), and there were a further two articles where this was possible but not certain from the text (32, 46). The highest reported seroprevalence recorded in these studies was 100.0% (36, 44) in goats and/or sheep in Oyo and Plateau states (sample size <10), or 76.2% in West African Dwarf goats in Eruwa, Oyo State (sample size = 42) (45) (Figure 2, Supplementary Table 1). Sample sizes for seroprevalence ranged considerably, from eight animals (36) to 4,548 animals (34) in total, and seven goats (44) to 3,489 goats (34), two sheep (44) to 1,059 sheep (34), 108 camels (8) to 1,517 camels (7), 192 cattle (8), 17 gazelle (*Dorcas gazelles*) (32), and 38 African gray duiker (*Sylvicapra grimmia*) (33). Seroprevalence in goats ranged from 22.2 to 100.0% (median: 54.5%, number of studies = 15), in sheep: 0.0%−100.0% (median = 45.4%, *n* = 9), in camels 0.0%−27.8% (median: 10.9%, *n* = 4), in cattle: 16.7% (*n* = 1) and in wild ruminants: 10.5%−76.5% (*n* = 2). Two studies, one focusing on camels in Borno and Kano states (sample size = 136) (31), and one in sheep in Oyo state (sample size = 5) (45) reported a seroprevalence of 0.0%.

Seven studies compared PPRV seroprevalence between male and female animals (6, 7, 15, 47, 49–51). In goats (*n* = 5 studies) seroprevalence for males ranged from 8.2 to 63.2% and for females 36.9%−81.3%; in sheep (*n* = 3) for males 20.5%−60.0% and for females 41.9% - 60.0%; and in camels (*n* = 2) for males 3.3%−10.3% and for females 3.6%−25.4%.

### Molecular Epidemiology

**Figure 3:**
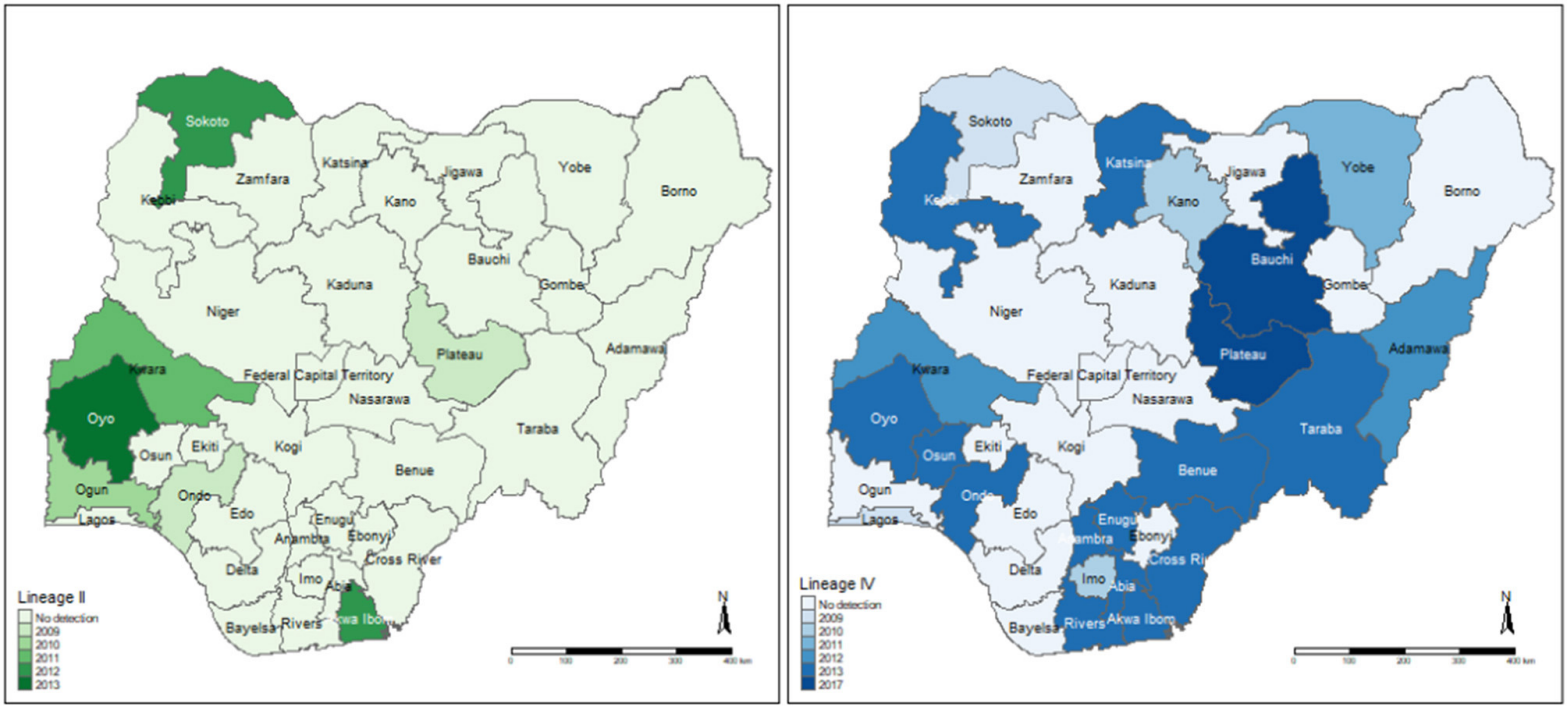
Locations of PPRV Lineage II and IV detection identified by the eligible articles. The year of the most recent detection per state is highlighted. Lineages may also have been identified previously in that state. The detection of lineage I by sequencing the *F* gene is not included as this method is no longer recommended for PPRV phylogenetics.

A total of 7/37 (18.9%) articles performed sequencing to identify PPRV lineages circulating at the time of the study (Supplementary Table 1). All sequenced isolates were collected from sheep and/or goats. One study (30) did not report sequencing results; instead, the accession numbers of the sequences on GenBank were reported. One study (62) reported that the nine samples sequenced from Kaduna and Plateau States in 2009 clustered into lineage I (based on phylogenetic analysis of *F* gene sequences), and had between 93 and 95% nucleotide similarity with the vaccine strain Nig 75/1 and Nig 76/1 (Figure 3). However, if the preferred method of sequencing the *N* gene had been performed, this isolate would have been grouped into lineage II. The remaining articles determined the lineage based on phylogenetic analysis of N gene sequences. Four articles (37–39, 42) reported lineage II in Sokoto, Plateau, Kwara, Ogun, Ondo and Akwa Ibom and Oyo States between 2010 and 2018. Five articles reported lineage IV (37–39, 42, 44) (Figure 3) in 22/37 states of Nigeria, between 2010 and 2020. Woma et al. (37) reported that lineage II isolates (*n* = 7) were subdivided into two distinct clades: II-NigA, closely related to PPRV viruses found in Mali, and II-NigB, closely related to the Nigeria 75/1 vaccine strain. Lineage IV isolates (*n* = 26) identified in the same study (37) were also subdivided into two clades: IV-NigA and IV-NigB.

### Risk Factors

A total of 10/37 (27.0%) articles reported risk factors associated with PPRV seropositivity using statistical analyses (Table 3). The most reported risk factor was age (*n* = 8 articles), followed by sex (*n* = 7), location (*n* = 5), species (*n* = 3), breed of goat (*n* = 1), body condition score (*n* = 1) and month of sampling (*n* = 1). All the articles mentioning sex as a risk factor observed a higher seroprevalence in female animals, in goats and sheep. Differing results were observed for both age and species as risk factors for PPRV seropositivity. A higher seroprevalence was observed in the oldest age group tested in 4/8 articles (6, 8, 49, 51), in the youngest age group in 3/8 articles (7, 32, 50), and in the mid-level age group in 1/8 article (48). When comparing goats with sheep, 2/3 articles observed a higher seroprevalence in goats (32, 49), while 1/3 observed a higher seroprevalence in sheep (48). Additionally, Red Sokoto goats (when compared with Sahelian and mixed breeds), a poor body condition score, and sampling animals in March, were associated with PPRV seropositivity, in three studies (7, 47, 48).

**Table 3:** Risk factors associated with PPRV seropositivity considered as statistically significant by the eligible articles.

**Risk factors identified**	***N***	**Study species**	**Details**	**Statistical method**	**Reference**
Age	8	Goats	>1 year/0–6 months/7 months−1 year	Odds ratio	(51)
		Goats, sheep	>2 years/ <1 year/1–2 years	Student *t*-test	(8)
		Goats, sheep	>3 years/ <1 year/1 <2 years/2 <3 years	Not specified	(49)
		Camels	>4 years/0–4 years	Chi-square test and Odds ratio	(6)
		Camels	0–5 years/>5 years	Chi-square test	(7)
		Goats, sheep, gazelles	Young/adult	Chi-square test	(32)
		Goats, sheep	1–2 years/ <1 year/>2 years	Chi-square test and Odds ratio	(48)
		Goats	<18 months/>18 months	Chi-square test	(50)
Sex	7	Goats	Females/males	Odds ratio	(51)
		Goats, sheep	Females/males	Student *t*-test	(8)
		Goats, sheep	Females/males	Not specified	(49)
		Camels	Females/males	Chi-square test and Odds ratio	(6)
		Goats, sheep	Females/males	Chi-square test	(32)
		Goats, sheep	Females/males	Chi-square test and Odds ratio	(48)
		Goats	Females/males	Chi-square test	(50)
Location	5	Goats	LGA: Gamawa/Boggoru	Odds ratio	(51)
		Sheep	LGA: *n* = 7	Not specified	(49)
		Goats, sheep	State: *n* = 12	Chi-square test	(34)
		Goats, sheep	LGA: *n* = 6	Chi-square test and Odds ratio	(48)
		Goats, sheep	State: Enugu**/**Anambra/Ebonyi	Chi-square test	(47)
Species	3	n/a	Goats/sheep	Not specified	(49)
			Gazelles/sheep	Chi-square test	(32)
			Goats/sheep	Chi-square test	(32)
			Sheep/goats	Chi-square test and Odds ratio	(48)
Breed of goat	1	Goats	Red Sokoto goats/Sahelian/Mixed	Chi-square test and Odds ratio	(48)
Body score	1	Camels	Poor body condition score/fair/good	Chi-square test	(7)
Month of sampling	1	Goats, sheep	March/December/January/February/April/ May/June	Chi-square test	(47)

*Underlined factors represent where there was a significantly increased/higher seropositivity*.

*N, number of articles identifying that risk factor; LGA, local government area*.

The studies used a variety of statistical methods including the Student *t*-test (*n* = 1), Chi-square test (*n* = 8) and the calculation of Odds Ratio (*n* = 3), and all considered a *p* value of 0.05 for significance. One article did not specify the statistical methods used (49). Two articles identified risk factors associated with the detection of PPRV antigen (implying current infection at the time of sampling), rather than seropositivity (a history of infection). These studies identified a higher incidence of PPRV in West African Dwarf Goats (54) and during the late wet and late dry seasons (53). Additionally, one article identified several risk factors associated with PPR cases as determined by a farmer questionnaire (61) using both univariate and multivariate analysis. In this study, more cases were observed in the dry season, in animals <12 months of age, in female animals and in goats.

## Discussion

This review considered 37 published research reports to summarize present knowledge of the epidemiology of PPR in Nigeria. Studies were distributed amongst most states and regions of Nigeria although no studies were found with respect to some of the Northern States (Zamfara and Jigawa), the middle belt (Federal Capital territory and Nasawara), and in or near the Niger Delta region (Delta, Edo and Ekiti). The reasons for this disparity in study regions are unclear but may be due to difficulty of access in certain locations; locations of laboratories, for example the location of the National Veterinary Research Institute, in Vom, Plateau State, which was one of the most visited; or due to political insecurity in some areas such as in the North Eastern States. Future research could consider including these areas if feasible, especially the North of Nigeria, particularly Zamfara and Jigawa states, which has a high small ruminant population (63).

Approximately half of the articles aimed to determine PPRV seroprevalence, with most studies focusing on small ruminants, although several studies also estimated PPRV seroprevalence in camels, cattle, and wild ruminants. A high seroprevalence for PPRV in small ruminants was reported in those regions of Nigeria that were studied, however it is noteworthy that almost half of the seroprevalence studies reviewed were conducted in the North East zone. There was a wide range of sample sizes for the seroprevalence studies reviewed, with three of the studies each sampling over 1,000 small ruminants: in North-eastern Nigeria (8), Bauchi State and Gombe State (32) and across the six different agro-ecological zones of Nigeria (34). Two of these large studies reported a seroprevalence of more than 50% (8, 32) in small ruminants with no previous history of vaccination, indicating the extent of this disease in the targeted states. Bello et al. (32) reported a higher seroprevalence in goats (73.8%) than sheep (19.4%), while El-Yuguda et al. (8) reported a higher seroprevalence in sheep (76.5%) than goats (51.6%). It is important to note that not all studies described in detail how animals were selected for sampling; sample size alone does not necessarily reflect the validity of a seroprevalence result due to the likelihood of herd/flock-level clustering. Additionally, these varying reports in seroprevalence in sheep compared to goats in these cross-sectional studies may reflect many unreported factors aside from species susceptibility, such as time since infection, grazing and housing management, and age structure of the flock. It is also important to note that four of the articles that reported seroprevalence did so with a link to an outbreak investigated within the same study, consequently biasing seroprevalence estimates. Information regarding seroprevalence in other West African countries is limited, highlighting the necessity for epidemiological studies to determine the extent of PPR infection throughout West Africa, particularly considering the high seroprevalence estimates observed in Nigeria.

Three of the four studies in camels (6–8) revealed serological evidence of previous PPRV infection; with seroprevalence estimates ranging from 3.4 to 27.8%. Clinical PPRV infection and seroconversion in camels has been reported from other endemic regions in Africa, and the Middle-East, with clinical signs similar to those in small ruminants (64, 65). Clinical signs in camels were not reported in the studies investigated in this review. Antibodies to PPRV have been detected in camels and the possibility of asymptomatic infection in camels suggests a possible challenge to the control of the disease in Nigeria, particularly where there is transboundary movement of camels in the Sahel regions (6). Further studies are required to determine the seroprevalence of PPRV in camels in Nigeria and other West African countries to elucidate the potential role of camels in the transmission of PPRV in small ruminants.

Only one study tested cattle for antibodies against PPRV, where 16.7% of 192 cattle tested were seropositive. Cattle were not included in the search terms for this review; however, an additional search using cattle or synonymous terms did not identify any further publications from Nigeria. The role of cattle in the maintenance of PPRV circulation is unknown, however, several studies in East Africa have identified spillover infection from small ruminants in cattle (66, 67). For example, in 2011, Lembo et al. found that cattle living near sheep and goats in the Serengeti, Tanzania were positive for PPRV antibodies; some of the cattle were older and were alive during a known PPR outbreak, but some young cattle only existed when there were no known clinical cases in local vaccinated small ruminants. This suggests that cattle may be a useful species for sentinel surveillance where mass vaccination programs for small ruminants is ongoing (66).

PPRV seropositivity in wild ruminants including gazelle (*Dorcas gazelles*) (32) and African gray duiker (*Sylvicapra grimmia*) (33) was reported in 2/2 studies, suggesting the potential for circulation of the virus in wildlife in Nigeria. PPRV has recently been identified in several wildlife species in other countries, including gazelle in Sudan (68), and saiga antelope (*Saiga tatarica*), Siberian ibex (*Capra sibirica*) and goitered gazelle (*Gazella subgutturosa*) in Mongolia (69), with evidence of interspecies transmission. Ogunsanmi et al. suggested that wild small ruminants may have played a role in the original introduction of PPRV to Nigeria, through the hunting of wild small ruminants in the South West region (33, 70), however this should be interpreted with caution as recent studies suggest it is likely that most outbreaks in wild animals originate from small ruminants farmed nearby (71, 72). There is a need for serological and clinical surveillance of PPRV in wild ruminants to determine the prevalence of PPR, its effects on wildlife conservation and the possible role of these species in the transmission cycle of PPRV in small ruminants.

Several risk factors for PPRV seropositivity were reported in the reviewed studies including age, sex, location, and species. Results with respect to age and species were inconsistent between studies. This may have been due to differences in study locations, sample size and local history of exposure to PPRV. Most of the studies investigating risk factors for seropositivity used basic statistical methods such as the Chi-squared test, calculation of odds ratio, or the student's *t*-test. Whilst these methods are useful, none of these studies performed multi-variable analysis, to allow for the assessment of potential confounding factors, so results must be considered with caution. Additionally, the methods of reporting results regarding risk factors and seroprevalence were inconsistent; some studies did not provide information such as sample numbers per variable, confidence intervals and *p*-values. These inconsistencies in study design and data reporting precluded any attempt at combining results across studies, for example by meta-analysis.

Notwithstanding, half of the studies that identified age as a significant risk factor found a higher seropositivity was associated with animals in their oldest age category, and this is likely due to the animals having had a longer time frame in which to become infected and recover. There is also the possibility that some of these animals may have been vaccinated but appropriate record keeping was lacking. The serological tests that were used do not distinguish between previously infected or vaccinated animals. Younger, immunologically naïve animals are more likely to be clinically affected and therefore these groups could be specifically targeted by vaccination campaigns to prevent outbreaks. Interestingly, for those studies where sex was identified as a risk factor (*n* = 7), all studies were in agreement, with female animals more likely to be associated with PPRV seropositivity. This is likely due to management practices, particularly of small ruminants where males are kept in the flock for short periods and sold for meat at 1–2 years of age, therefore have less time for PPRV exposure. Indeed, several of the studies did not provide information on the proportions of male and female animals sampled, although interestingly, one study did report sampling almost twice as many male animals. Unfortunately, these studies did not report an age-adjusted seroprevalence and it could not be estimated from the available data. Diverse small ruminant management and production systems are operated across Nigeria, which may influence the incidence of PPR. Furthermore, seasonal and annual variations in climate may further impact the occurrence of infection. Therefore, whilst some authors observed a higher seroprevalence in some areas, it is unsurprising that overall, this review did not discern any consistent evidence of areas with a higher seroprevalence.

Approximately half of the studies investigated current or retrospective outbreaks of PPRV, using several methods for diagnosis including clinical signs with post-mortem, and/or the use of confirmatory antigen-based or molecular tests. All authors that observed clinical disease reported high morbidity, a typical feature of PPR. Molecular techniques were used by eight of the studies and these techniques have advantages over traditional antigen detection tests in that they are highly sensitive, high throughput and rapid. However, molecular techniques do not identify whether virus is infectious or viable, in comparison to techniques such as virus isolation or agar gel immunodiffusion (13). In one study, Chukwudi et al. (30) compared a colorimetric RT-LAMP assay to RT-PCR using samples collected from sheep and goats. RT-LAMP has the potential for field application, as the method does not rely on thermal cycling, so can be performed in a water bath, heat block, or, where the assay relies on fluorescent detection, using specific portable equipment (73, 74). Rapid pen-side diagnostics, such as immunochromatographic lateral flow devices that can give a result in less than 30 min (75), were not utilized in any of the reviewed studies, which may suggest that they are not widely employed in Nigeria. The deployment of these rapid techniques may be particularly useful in remote settings far from centralized testing laboratories, to speed up diagnosis and the implementation of control measures (76). Additionally, further investigation into the utility of alternative, non-invasive sample types such as feces and milk for surveillance should be considered (77, 78).

Molecular characterization and phylogenetic analysis to identify the circulating PPRV lineages was performed in six studies on samples collected from sheep and/or goats. All but one of these analyses were performed based based on *N* gene sequences, currently the preferred method as it is more suitable for phylogenetic distinction between closely related circulating viruses (26, 79). Only one article performed phylogenetic analysis based on *F* gene sequences, on isolates collected in 2009 (62). Based on this method, these isolates clustered into lineage I (62), however, sequencing of the *N* gene, as reported in the other reviewed studies, would group this isolate into lineage II. These *F*-gene based sequences (62) had a similarity to the vaccine strain, Nigeria 75/1, which was first isolated from Nigeria in an outbreak in 1975 and has since been distributed in West Africa (80). Several other studies also identified isolates with sequences closely related to the Nigeria 75/1 vaccine strain, grouped into lineage II according to *N* gene phylogenetic analysis, the most recent being in 2013 (37, 39, 42). It has been reported that those isolates with sequences that have a high similarity to the vaccine strain, may have been contaminated in the laboratory (81, 82), and therefore the likelihood of this strain still circulating in Nigeria should be treated with caution, with further studies required. Interestingly, Woma et al. (37) and Mantip et al. (38) identified sequences in 2011/12 (II-NigA) and 2018, respectively, which were well separated from those previously reported sequences similar to vaccine strain Nigeria 75/1. The sequences were distinct, suggesting a transboundary circulation of genetically diverse PPRV strains both in Nigeria and in neighboring countries. Lineage III was not detected in any of the studies, which is expected considering this lineage is more widespread in East Africa and part of the Middle East (26, 83).

Lineage IV historically existed in Asia; however, it has since extended west into Africa, where it has the potential to replace other PPRV viral lineages (26, 80, 83). This is supported by results of this review, where five of the articles reported the detection of lineage IV (37–39, 42, 44) between 2010 and 2020, including the first reports of this lineage circulating in Nigeria (37, 39). Lineage IV viruses were identified in 22/37 states of Nigeria, highlighting how widespread this lineage has become in Nigeria. Geographical clustering of sequences suggests high diversity, and similarities with sequences from neighboring countries such as Niger indicate likely spread of the virus across borders (38, 39). Further molecular epidemiology studies, particularly focusing on regions where studies are lacking, would assist in obtaining a fuller picture of disease circulation and monitoring of transboundary movements of the disease. Additionally, using sequence information already available to trace outbreaks, may help to inform future control efforts.

Vaccination is a highly effective tool for PPR control, as live attenuated PPR vaccines can induce lifelong protective immunity, and unlike for many other diseases such as foot-and-mouth disease, PPR vaccines are thought to be efficacious against all known serotypes and lineages (3, 84, 85). However, results of this review demonstrate that PPR is still a major problem in Nigeria, and outbreaks have continued to occur in the country during the last 20 years, despite the availability of a nationally produced homologous PPRV vaccine (86). Only four of the reviewed articles mentioned that PPRV vaccination was performed in the study area at the time of sampling, potentially highlighting the low vaccination coverage in Nigeria (87), although this is based on limited evidence from the reviewed studies, and consequently vaccination uptake in Nigeria needs to be further investigated. One older study mentioned vaccination against rinderpest (54), which also provides some protection against PPRV infection. Several of the seroprevalence studies specifically identified farms where no vaccination had occurred. In animals previously vaccinated with the homologous PPRV Nig 75/1 vaccine it is not currently possible to use serological methods to differentiate between a history of either PPRV infection or vaccination (DIVA) (48), and therefore this may have biased results. Additionally, it is plausible that record keeping on farms is poor, and therefore vaccination history is unknown, particularly when purchasing new stock from markets. When performing structured interviews to farmers and veterinarians, Chukwudi et al. (47) identified that there was a lack of awareness of PPR vaccination among farmers, a lack of availability of PPR vaccine to veterinarians, and that there was limited use of veterinary services by sheep and goat owners.

Vaccination strategies are influenced by several factors including socioeconomic factors and turnover of the small ruminant population. Socioeconomic factors can influence the effectiveness of disease control strategies such as a PPR vaccine program (62, 88), and it is recognized that good quality veterinary services, training of personnel, maintenance of a cold chain for vaccine storage and transportation (3, 17, 47, 89) are essential. Additionally, because there is a high turnover of the small ruminant population, timing and frequency of vaccination is crucial to ensure enough animals are protected to enable herd immunity, for which the threshold is estimated at approximately 80% (3, 90). Consequently, despite the availability of an effective vaccine, the aim of eradication of PPR is likely more challenging than that of rinderpest virus due to the longer lifespan of cattle and their higher economic importance (18).

If PPR is to be eradicated in Nigeria by 2030 in alignment with the PPR global eradication strategy (3), there is a need for improvement of the veterinary infrastructure, improvement of farmer awareness, disease diagnosis, training, and surveillance. The use of thermostable vaccines, which have been trialed in Nigeria with good results in the field (89), may enable vaccination of animals where cold storage is a problem, and those with DIVA capability would assist post-vaccination sero-surveillance activities, which were used successfully during the eradication of rinderpest virus to monitor the success of vaccination programs in the field (26). Additionally, stricter implementation of record keeping, quarantine, control of animal movement, may also assist in reducing the burden of PPR in livestock and wildlife in Nigeria (18, 27).

When performing this review, the authors observed that several articles lacked detail with respect to study design, despite these studies fitting the inclusion criteria. Additionally, some studies did not provide full details on all variables. These issues precluded a formal meta-analysis. However, it was considered that stricter inclusion criteria would have greatly diminished the range of evidence to inform this study. In summary, this review has highlighted the high prevalence of PPRV in Nigeria and identified regions of Nigeria where epidemiological data is not available/accessible. Information on the economic impact of PPR and its eradication in Nigeria is lacking, in addition to the reasons for low uptake of the PPRV vaccine. Performing studies to investigate these topics may help to understand the awareness and knowledge of farmers and veterinary health professionals regarding PPR prevalence and the benefits and barriers to PPR control efforts and may provide evidence to support decision-making by key stakeholders and governmental bodies.

## Data Availability Statement

The original contributions presented in the study are included in the article/Supplementary Material, further inquiries can be directed to the corresponding author.

## Author Contributions

AE, RA, BA, DE, and MB participated in the design of the study. DE, BA, and MB were involved in systematic review, data analysis and interpretation. DE and BA were involved in preparation of the manuscript. All authors have read, contributed, and approved the final manuscript.

## Funding

This study was supported by the African Livestock Productivity and Health Advancement (ALPHA) Initiative, co-funded by the Bill and Melinda Gates Foundation (BMGF) and Zoetis. Funding from Zoetis was an unrestricted grant. BMGF Grant Number: OPP1165393.

## Conflict of Interest

The authors declare that the research was conducted in the absence of any commercial or financial relationships that could be construed as a potential conflict of interest.

## Publisher's Note

All claims expressed in this article are solely those of the authors and do not necessarily represent those of their affiliated organizations, or those of the publisher, the editors and the reviewers. Any product that may be evaluated in this article, or claim that may be made by its manufacturer, is not guaranteed or endorsed by the publisher.
