# Supplementary material for: Epidemiology of Peste des Petits Ruminants in Nigeria: A Review
Source: Front Vet Sci. 2022 Jul 6;9:898485. doi: 10.3389/fvets.2022.898485 (PMC9298765; doi:10.3389/fvets.2022.898485)
Supplement: Supplementary file 1 [file Table_1.DOCX]

**Supplementary Data Table 1**. Data extracted from the 37 eligible articles.

| **Title** | **Authors/date** | **Study areas** | **Diagnostic method** | **Sero-prevalence** | **Disease prevalence/ antigen detection** | **Lineage detected** |
| --- | --- | --- | --- | --- | --- | --- |
| Comparative study of some prevalent diseases of ecotype goats reared in southeastern Nigeria | Odo, 2003 | Abakaliki, Ebonyi State | Clinical history,  HA |  | Maximum annual prevalence over 5-year period:  Red Sokoto goats 10.0%,  crossbreed goats 8.0%,  West African Dwarf goats 18.0% | N/A |
| Incidence and modulating effects of environmental factors on trypanosomosis, peste des petit ruminants (PPR) and bronchopneumonia of West African dwarf goats in Imo state, Nigeria | Okoli, 2003 | Imo State | Clinical history, AGID |  | Total: 19.5% (6714/26763),  1999: 17.5% (1762/10090),  2000: 17.8% (1784/10018),  2001: 25.2% (1679/6657) | N/A |
| Peste des petits ruminants (PPR) virus antibodies in African grey duiker (*Sylvicapra grimmia*) | Ogunsanmi *et al.,* 2003 | Osun State | cELISA | 10.5% (4/38) |  | N/A |
| Studies on the prevalence of antibodies to peste des petits ruminants virus (PPRV) among goats in Bauchi State | Luther *et al.,* 2006 | Bauchi State | cELISA | Total: 24.7% (56/227),  Gamawa LGA: 43.1%,  Boggoru: 10.3%,  Males: 8.2%,  Females: 36.9% |  | N/A |
| Pest of small ruminants as a major constraint to small ruminant production in Cross River State, Nigeria | Owai, 2007 | Cross River State | Clinical history,  post-mortem |  | Total: 7.1% (141/1981),  1996: 7.6% (25/331),  1997: 6.3% (56/894),  1998: (13.0%) (43/330),  1999: 4.0% (17/426) | N/A |
| Evaluation of peste des petits ruminant and Rinderpest virus infection of camels in Borno and Kano states of Nigeria | Ibu *et al.*, 2008 | Borno, Kano States | Immunocapture ELISA (ICE), cELISA | Total: 0.0% (0/136) | Total: 0.0% (0/136) | N/A |
| Prevalence of PPR cases among Sahel goats presented at the Borno State Veterinary Clinic Maiduguri Nigeria from 1996-2005 | El-Yuguda *et al*., 2008 | Maiduguri, Borno State | Clinical history |  | 1999: 36.5%,  Rainy season: 47.7%,  Harmattan season: 28.2%,  Hot dry season: 24.1%,  Females: 63.9%,  Males: 36.1%,  Adult goats: 81.2%,  Kids: 18.8%. | N/A |
| Outbreak of peste des petits ruminant in an unvaccinated Sahel goat farm in Maiduguri, Nigeria | El-Yuguda *et al.,* 2009 | Maiduguri, Borno State | AGID,  VI,  VNT*,  clinical history,  post-mortem | Total: 72.9% (97/133),  Goats: 75.2% (85/113),  Sheep: 60.0% (12/20), |  | N/A |
| Peste Des Petits Ruminants Virus (PPRV) Infection Among Small Ruminants Slaughtered at the Central Abattoir, Maiduguri, Nigeria | El-Yuguda *et al*., 2009 | Maiduguri, Borno State | AGID |  | Sheep: 41.0%,  Goats: 37.7% | N/A |
| The current status of Peste des Petits Ruminant (PPR) in sheep in Ibadan southwestern Nigeria. | Jagun and Onoja, 2011 | Ibadan, Oyo state | HI*,  VI,  Clinical history,  Post-mortem | Total: 73.0% (146/200),  West African dwarf sheep: 77.5% (124/160),  Yankasa sheep: 55.0% (22/40) |  | N/A |
| Molecular characterization and phylogenetic study of peste des petits ruminants viruses from north central States of Nigeria | Luka *et al.,* 2011 | Kaduna and Plateau state | RT-PCR, sequencing (F gene) |  | Total: 51.5% (17/33),  Kaduna: 100.0% (7/7),  Plateau: 38.5% (10/26) | Lineage 1 (n=9) |
| Outbreak of Peste Des Petits Ruminants in West African Dwarf Goats in Eruwa, Southwestern Nigeria | Lawal *et al*., 2011 | Ibadan, Oyo State | Clinical examination, cELISA | Total: 76.2 (32/42),  Clinically affected goats: 75.0% (15/20),  Recovered goats: 100.0% (10/10),  Apparently healthy goats: 100.0% (7/7),  Apparently healthy sheep: 0.0% (0/5) |  | N/A |
| Molecular diagnosis of recent suspected outbreaks of peste des petits ruminants (PPR) in Yola, Adamawa State, Nigeria | Ularamu *et al*., 2012 | Yola, Adamawa State | RT-PCR |  | Total: 27.3% (9/33) | N/A |
| Seroprevalence of peste des petits ruminants among domestic small and large ruminants in the semi-arid region of north-eastern Nigeria | El-Yuguda *et al.,* 2013 | Semi-arid region of North-eastern Nigeria | VNT*,  c- ELISA | (n = 2879, goats = 1571 sheep = 1008, camels = 108, cattle = 192)  57.0% using VNT,  55.0% using c-ELISA,  *Sheep: 76.5%,  *Goat: 51.6%,  *Camel: 27.8%  *Cattle: 16.7% |  | N/A |
| Seroprevalence of Peste Des Petits Ruminants among Goats and Sheep in Enugu State of Nigeria. | Nwobodo *et al.,* 2013 | Enugu State | cELISA | (n = 655, goats = 429 sheep = 226)  Total: 47.0%,  Enugu-East: 38.9%,  Enugu- Ezike: 57.0%,  Goats: 53.6%,  Sheep: 34.5%,  Males: 33.0%,  Females: 56.0% |  | N/A |
| Goat management systems and peste des petits ruminant (PPR) incidence in rivers and Bayelsa states, Nigeria. | Okpeku *et al.,* 2013 | Rivers State and Bayelsa State | Clinical history |  | Maximum Rivers State: 22.0%, Maximum Bayelsa: State 26.0% | N/A |
| Retrospective Study of Disease Incidence and Type of Pneumonia in Nigerian Small Ruminants in Ibadan, Nigeria | Emikpe *et al*., 2013 | Ibadan, Oyo State | Clinical history,  post-mortem |  | Total: 71.4% (75/105) | N/A |
| Studies on Influence of Seasonality on Clinical Conditions of Small Ruminants in Ogbomoso Areas of Oyo State | Ameen and Ajayi, 2013 | Ogbomoso, Oyo State | Clinical history,  post-mortem |  | Total: 7.7% (66/859),  late dry season: 9.6% (21/219), early dry season: 6.3% (13/206), early wet season: 7.0% (15/215), late wet season: 7.2% (17/219) | N/A |
| Enzyme Linked Immunosorbent Assay (ELISA) Based Detection of Antibodies to Peste Des Petits Ruminants Virus in Camels Presented for Slaughter at Sokoto Municipal Abattoir, Northwestern Nigeria | Bello *et al.,* 2013 | Sokoto State | c-ELISA | Total: 18.3% (25/137),  Females: 25.4% (18/71),  Males: 10.3% (7/66) |  | N/A |
| Serological evidence of camel exposure to peste des petits ruminants virus (PPRV) in Nigeria | Woma *et al*., 2015 | Northern Nigeria | c-ELISA | Total: 3.4% (51/1517),  Borno: 3.0% (13/433),  Kano: 3.3% (17/517),  Katsina: 4.1% (12/296),  Sokoto: 3.3% (9/271),  Males: 3.3% (32/984),  Females: 3.6% (19/532) |  | N/A |
| Co-circulation of Peste-des-Petits-Ruminants Virus Asian lineage IV with Lineage II in Nigeria | Woma *et al*., 2015 | Six different agro-ecological zones of Nigeria | RT-PCR,  Sequencing (N gene) |  | Total: 42.0% (33/79),  Sheep: 12.5% (2/16),  Goats: 49.2% (31/63) | Lineage II (n=7) and lineage IV (n=26) |
| Molecular analysis of peste des petits ruminants viruses from current outbreaks in Nigeria | Woma *et al.*, 2015 | Six different agro-ecological zones of Nigeria | RT-PCR,  Sequencing (N gene) |  | Total: 57.9% (81/140) | Lineage II and lineage IV |
| Research for Peste des Petits Ruminants (PPR) Virus Antibodies in Goats, Sheep and Gazelle from Bauchi and Gombe States, North Eastern Nigeria | Bello *et al.,* 2016 | Bauchi State and Gombe State | cELISA | Total: 61.1% (2579/4223),  Goats: 73.8% (2375/3218),  Sheep: 19.4% (192/988),  Gazelle: 76.5% (13/17),  Bauchi: 60.4%,  Gombe: 61.7% |  | N/A |
| Serosurvey of peste des petits ruminants virus in small ruminants from diﬀerent agro-ecological zones of Nigeria | Woma *et al*., 2016 | Agro-ecological zones of Nigeria | c-ELISA | Total: 22.4% (1018/4548),  Goats: 22.2% (744/3489),  Sheep: 23.0% (244/1059),  Adamawa: 20.5%,  Taraba: 29.5%,  Anambra 19.9%,  Imo: 28.3%,  Akwa Ibom: 25.9%,  Cross River: 14.5%,  Plateau: 21.4%,  Kwara: 28.0%,  Kano: 30.1%,  Sokoto: 22.9%,  Ogun: 17.8%,  Ondo: 19.0%,  Males: 22.6%,  Females: 23.6% |  | N/A |
| Comparison of nucleotide sequences of recent and previous lineages of peste-des-petits-ruminants viruses of sheep and goats in Nigeria | Mantip *et al*., 2016 | 18 states, three states of each of the six agro-ecological zones | RT-PCR,  Sequencing (N gene) |  | Total: 9.7% (35/360) | Lineage II and lineage IV |
| Field evaluation and confirmation of acute peste des petits ruminant outbreak in a flock of West African dwarf goats in Ibadan, Nigeria | Adeola *et al*., 2017 | Ibadan, Oyo State | Clinical history,  c- ELISA,  post-mortem examination | c-ELISA: 100% (8/8) | Morbidity: 96.0% (24/25),  Mortality: 60.0% (15/25), | N/A |
| Risk Factors Associated with Peste des Petits Ruminants (PPR) in Sheep and Goats in Makurdi, Benue State | Victor *et al*., 2017 | Makurdi, Benue State | Clinical history |  | Total: 51.3% (78/152),  Sheep: 35.1% (13/37),  Goats: 56.5% (65/115),  North-Bank: 64.0% (32/50),  Wurukum: 60.0% (18/30),  Wadata: 36.4% (12/33),  Modern Market/Ankpa Ward: 41.0% (16/39) | N/A |
| First report of immunohistochemical detection of Peste des petit ruminants, parainfluenza 3 and respiratory syncytial viral antigens in lungs of Nigerian goats | Jarikre and Emikpe, 2017 | Ibadan, Oyo state | Immunohistochemical detection of PPR antigen |  | 34.0% (51/150) | N/A |
| Prevalence of suspected peste des petits ruminants infection and complicating bacteria in goats in Abeokuta, Ogun State, Nigeria | Okwelum *et al.*, 2017 | Abeokuta, Ogun State | Clinical history,  post-mortem |  | 6/6 herds | N/A |
| Seroprevalence of peste des petits ruminants among unvaccinated small ruminants in Sokoto State, northwestern Nigeria | Bello *et al.,* 2018 | Sokoto State | cELISA | Total: 45.5% (197/433),  Sheep: 52.4% (98/187),  Goats: 40.2% (99/246),  Sokoto South: 68.6%,  Tambuwal: 59.1%,  Goronyo: 56.7%,  Kware: 53.2%,  Bodinga: 33.2%,  Wurno: 18.6% |  | N/A |
| Peste Des Petits Ruminants Infection and Infertility in West African Dwarf (WAD) Goats in Makurdi, Benue State, Nigeria | Wachida *et al*., 2018 | Makurdi, Benue State | Clinical history, blocking ELISA | Total: 37.3% (44/118) |  | N/A |
| Co-infection of peste des petits ruminants and goat pox in a mixed flock of sheep and goats in Kanam, North Central Nigeria | Adedeji *et al.,* 2019 | Kanam, Plateau State, North Central Nigeria | c-ELISA,  RT-PCR,  Sequencing (N gene) | 100.0% (9/9) using c-ELISA,  Goats: 100.0% (7/7),  Sheep: 100.0% (2/2) | 100.0% (1/1) using RT-PCR | Lineage IV |
| The First Seroprevalence Investigation of Peste Des Petits Ruminants Virus among Sahel Goat in Yobe State, Nigeria | Bukar *et al.,* 2020 | Yobe state | c-ELISA | Total: 55.4% (255/460),  Bursari: 56.1% (87/155),  Bade: 55.4% (97/175),  Nangere: 54.6% (71/130),  Males: 44.4%,  Females: 60.0% |  |  |
| Update on Peste des petits ruminants status in South East Nigeria: serological and farmers’ awareness investigation, and potential risk factors | Chukwudi *et al.,* 2020 | Enugu, Anambra and Ebonyi States, South-East Nigeria | cELISA | Total: 42.5% (121/285),  Goats: 38.1%,  Sheep: 45.4%,  Enugu: 62.2%,  Anambra: 34.8%,  Ebonyi: 20.3% |  | N/A |
| Comparison of colorimetric loop-mediated isothermal amplification kit and reverse transcription-polymerase chain reaction in the diagnosis of peste des petits ruminants in sheep and goats in Southeast Nigeria | Chukwudi *et al.*, 2020 | South-East Nigeria | RT-PCR,  colorimetric RT-LAMP,  sequencing |  | 29.5% (65/220) | N/A |
| Peste Des Petits Ruminants Virus and Goat pox Virus Co-Infection in Goats | Akanbi *et al*., 2020 | Kanam, Plateau State, North Central Nigeria | histopathology, transmission electron microscopy |  | 100.0% (1/1) | N/A |
| Molecular epidemiology of peste des petits ruminants virus in Nigeria: an update | Mantip et al., 2021 | 18 states | RT-PCR, sequencing (N gene) |  | 23.1% (99/429) | Lineage IV (n=90) and  lineage II (n=1) |

c-ELISA: competitive-enzyme-linked immunosorbent assay, RT-PCR: reverse transcription polymerase chain reaction, AGID: Agar gel immunodiffusion test, HA/HI Haemagglutination/ Haemagglutination inhibition test, VNT: virus neutralization test, VI: virus isolation, RT-LAMP: reverse transcription loop-mediated isothermal amplification.

*Indicates the test used for prevalence results if more than one test/measurement used in the study.
